# A Novel Combinatorial Epigenetic Therapy Using Resveratrol and Pterostilbene for Restoring Estrogen Receptor-α (ERα) Expression in ERα-Negative Breast Cancer Cells

**DOI:** 10.1371/journal.pone.0155057

**Published:** 2016-05-09

**Authors:** Rishabh Kala, Trygve O. Tollefsbol

**Affiliations:** 1 Department of Biology, University of Alabama Birmingham, 1300 University Boulevard, Birmingham, Alabama, 35294, United States of America; 2 Comprehensive Center for Healthy Aging, University of Alabama Birmingham, 1530 3rd Avenue South, Birmingham, Alabama, 35294, United States of America; 3 Comprehensive Cancer Center, University of Alabama Birmingham, 1802 6th Avenue South, Birmingham, Alabama, 35294, United States of America; 4 Nutrition Obesity Research Center, University of Alabama Birmingham, 1675 University Boulevard, Birmingham, Alabama, 35294, United States of America; 5 Comprehensive Diabetes Center, University of Alabama Birmingham, 1825 University Boulevard, Birmingham, Alabama, 35294, United States of America; University of Navarra, SPAIN

## Abstract

Breast cancer is the second most common cancer and a leading cause of cancer death in women. Specifically, estrogen receptor-α (ERα)-negative breast cancers are clinically more aggressive and normally do not respond to conventional hormone-directed therapies such as tamoxifen. Although epigenetic-based therapies such as 5-aza-2’-deoxycytidine and/or trichostatin A as DNA methyltransferase (DNMT) and histone deacetylase (HDAC) inhibitors, respectively, can regulate the expression of ERα, this can often lead to a number of side effects. Plant-based dietary compounds such as resveratrol and pterostilbene in novel combinatorial therapy provides new avenues to target these side effects and provide similar results with a higher level of safety. Here, we report that combinatorial resveratrol and pterostilbene leads to the reactivation of ERα expression in ERα-negative breast cancer cells in a time-dependent manner. Chromatin immunoprecipitation analysis of the *ERα* promoter in each cell type revealed an increase in enrichment of acetyl-H3, acetyl-H3lysine9 (H3K9) and acetyl-H4 active chromatin markers in the *ERα* promoter region after combinatorial treatment. This treatment also resulted in a significant change in HDAC and histone acetyl transferase (HAT) enzyme activity in these cells after 3 days of treatments. The combination resulted in a significant decrease in DNMT enzyme activity and 5-methylcytosine levels in MDA-MB-157 breast cancer cells. Moreover, reactivation of ERα expression by resveratrol combined with pterostilbene was found to sensitize ERα-dependent response to 17β-estradiol (E2)-mediated cellular proliferation and antagonist 4-hydroxytamoxifen (4-OHT)-mediated inhibition of cellular proliferation in ERα-negative breast cancer cells. E2 and 4-OHT further affected the ERα-responsive downstream *progesterone receptor* (*PGR*) gene in ERα reactivated MDA-MB-157 cells. Collectively, our findings provide a new and safer way of restoring ERα expression by regulating epigenetic mechanisms with the use of phytochemicals in combinatorial therapy. This combination can further provide effective treatment options for hormonal refractory breast cancer with available anti-hormonal therapy.

## Introduction

Breast cancer is the most frequently diagnosed cancer after skin cancer and the leading cause of cancer death among women in the United States after lung cancer [1]. Although the majority of breast cancers are ER-positive and approximately 25–30% are ER-negative, current chemotherapeutic strategies for breast cancer treatment commonly rely on the expression of important biomarkers such as estrogen receptor (ER), progesterone receptor (PGR) and HER2 protein [2,3]. Triple-negative breast cancers (TNBC), which typically lack these key biological markers, have a poor prognosis and are more resistant to hormone therapies in contrast to triple-positive breast cancers [4]. The status of ER plays an important role in monitoring the malignant behavior of breast cancer and can be targeted by known selective estrogen receptor modulators (SERMs) and/or with aromatase inhibitors (AIs). Of the two major isoforms of ERs (ERα and ERβ), ERα is found to be the primary contributor to estrogen induced growth-stimulatory effects in breast cancers [5].

It is important to recognize that in many cases the *ERα* gene expression in ER-negative breast cancer may not be attributed to DNA mutation, but rather acquired from epigenetic aberrations of the *ERα*promoter region in ERα-negative breast cancer cells [6, 7]. The common epigenetic aberrations are promoter hypermethylation and histone modifications, specifically histone deacetylations which are underlying mechanisms for ERα silencing in human malignant mammary cells [6, 8–12]. Therefore, many studies have reported the possibility of converting ER status through the use of epigenetic modulating drugs such as 5-aza and/or TSA leading to the reactivation of ERα expression, which in turn sensitizes these cells to current hormone therapies. One of the limitations with this current epigenetic approach is that it often leads to adverse side effects.

A number of growing studies have indicated that nutritional factors can play an important role in modulating human diseases, act as strong epigenetic modulators and have activities against cancers [12, 13]. Resveratrol (3, 5, 4’-trihydroxystilbene) and pterostilbene (3, 5-dimethoxy-4’-hydroxystilbene), are among the dietary phytoalexins synthesized by certain plants such as grapevines, peanuts and blueberries in response to an injury [12]. Both of the compounds when used together synergistically inhibit the cellular proliferation [4], and result in apoptosis induction and cell cycle arrest in TNBC cells. Recently, one potential mechanism that has received considerable attention is that resveratrol and pterostilbene when used together can modulate gene expression by influencing epigenetic processes such as DNA methylation, histone modification and/or DNA damage response (DDR) by targeting DNMTs and silence information regulatory enzyme (SIRT1), a type III HDAC. This decrease in SIRT1 and DNMT activity is also associated with a decrease in human telomerase reverse transcriptase (hTERT) expression and activity, which subsequently leads to tumor suppression [4, 14]. DNA methylation and histone acetylation have been the current focus in cancer prevention and therapy; however, this will be the first study observing the combinatorial epigenetic effects of resveratrol and pterostilbene at close to physiological achievable doses on *ER*α reexpression in TNBC cells.

In the present study, we tested our hypothesis that a combination of resveratrol and pterostilbene may lead to transcriptional activation of *ERα* expression in hormone-resistant breast cancer cells. Our study demonstrates that treatment of ER-negative breast cancer cells with resveratrol and pterostilbene can reactivate ERα expression through epigenetic modulation of DNA methylation and histone acetylation, specifically H3 and H4, which result in an open chromatin structure at the *ERα* promoter. Clinically, this reactivation of ER*α* by combinatorial dietary treatment enhances chemosensitivity in ER*α*-negative breast cancer cells towards tamoxifen, an anti-estrogen drug. This collectively illustrates the utility of combinatorial dietary compounds with current hormonal therapies to treat recalcitrant breast cancer.

## Methods

### Cell Lines

ERα-negative breast cancer cell lines MDA-MB-157, HCC1806 and ERα-positive breast cancer cell line MCF-7 were obtained from the American Type Culture Collection (ATCC, Manassas, VA). Clinically, MDA-MB-157 cells were initially obtained from a 44 year-old black female and HCC1806 cells were obtained from 60 year-old black female, hence allowing us to target two different age groups of breast cancers with ER-negative status. MCF-7 is an estrogen receptor-positive cell line which serves as the positive control for several of the experiments. Reported herein MCF10A is an immortalized, non-tumorigenic ERα-negative breast epithelial cell line used as a control in this study as commonly done [4, 14]. MDA-MB-157, HCC1806 and MCF10A cells were grown as reported previously [4]. MCF-7 cells were grown in DMEM media (Mediatech Inc, Manassas, VA) supplemented with 10% fetal bovine serum (Atlanta Biologicals, GA) and 1% penicillin/streptomycin (Mediatech). All of the cell lines were maintained in an incubator at 5% CO_2_ with a controlled temperature of 37°C.

### Chemicals

Resveratrol (>99% pure; HPLC; Cat # R5010) and pterostilbene (>97% pure; HPLC; Cat # P1499) were purchased from Sigma-Aldrich. The compounds were dissolved in dimethyl sulfoxide (DMSO) at a stock concentration of 50 mM and stored in dark at -20°C. Cells were treated with fresh dose of resveratrol (res) and pterostilbene (ptero) every 24 h (one day) for up to 72 h (three days) after seeding. DMSO was used as the vehicle control. For ERα responsive effects 4-Hydroxytamoxifen (4-OHT) which is ≥70% Z isomer and 17β-estradiol (E2) were purchased from sigma. 4-Hydroxytamoxifen is an active metabolite of tamoxifen.

### MTT analysis for cell viability

As previously published, the optimum dose of resveratrol and pterostilbene was used to determine the effects of ERα reactivation on cellular proliferation. ERα-negative MDA-MB-157 cells were plated in 96-well plates and were treated with the compounds singly as well as in combination for 72 h and then were exposed with 10 nM of 17β—estradiol (E2) (Sigma) and 5 μM of 4-hydroxytamoxifen (4-OHT) (sigma) for an extra two days (48 h). At the end of each treatment (72 h + 48 h), viability of cells was determined by performing MTT assay as published previously [4].

### Real-time PCR

To determine the change in expression of the *ERα* and *PGR* genes, Real-time PCR was performed. Breast cancer cells were treated as discussed previously. Total RNA was extracted using RNeasy Kit (Qiagen, Valencia, CA) and cDNA was prepared using cDNA synthesis kit (Bio-Rad). *ERα*forward primer: 5’- *GAACCGTCCGCAGCTCAAGATC*-3’ and reverse primer: 5’- *GTCTGACCGTAGACCTGCGCGTTG*-3’; *PGR* forward primer: 5’-*TCATTACCTCAGAAGATTTGTTTAATC*-3’ and reverse primer: 5’-*TGATCTATGCAGGACTAGACAA*-3’ and *GAPDH* forward 5’-*ACC ACA GTC CAT GCC ATC AC*-3’ and reverse primer: 5’-*TCC ACC CTG TTG CTG TA*-3’-were obtained from Integrated DNA Technologies, Inc. All the reactions were performed in triplicate and SYBR green Supermix (Bio-Rad) was used as fluorescent dye in Roche Light cycler 480. Thermal cycling was initiated at 94°C for 4 min followed by 35 cycles of PCR (94°C, 15s; 54°C, 30s; 72°C, 30s). GAPDH was used as an endogenous control and vehicle control was used as a calibrator. The relative changes in gene expression were calculated using the following formula: Fold change in gene expression, 2^-ΔΔCt^ = 2^-{ΔCt (treated samples)-ΔCt (untreated control samples)}^, where ΔCt = Ct (genes of interest)–Ct (GAPDH) and Ct represents threshold cycle number.

### Western Blotting

Protein extractions were performed using RIPA Lysis Buffer (Upstate Biotechnology, Charlottesville, VA) according to the manufacture’s protocol. After protein extraction, quantification was performed following Bradford method (Bio-Rad; Hercules, CA). About 50 μg of the whole cell protein extract were loaded onto a 4–15% Tris-HCL gel (Bio-Rad) and separated by electrophoresis at 200 V. Separated proteins were then transferred using Trans-Blot Turbo transfer system (Bio-Rad) at 25 V for 8 min. Membrane blocking were performed with 0.5% dry milk in Tris Buffered saline solution with 1% Tween (TBST) using SNAP i.d. protein detection system. Primary and secondary antibody incubation was carried out according to the manufacturer’s protocol. Membranes were probed with the monoclonal antibodies to ERα (6F11; NeoMarkers, Fermont,CA) and β-actin (Cell signaling). Immunoreactive bands were visualized using the Bio-Rad ChemiDoc XRS^+^ system.

### Chromatin Immunoprecipitation (ChIP) assay

MDA-MB-157 cells and HCC1806 TNBC cells were treated with 15 μM resveratrol or 5 μM pterostilbene alone or in combination for 48 h and 72 h. Approximately 2 × 10^6^ cells were crosslinked with a 1% final concentration of formaldehyde (37%, Fisher Chemicals, Fairlawn, NJ) for 10 min at 37°C. ChIP assays were performed with the EZ-Chromatin Immunoprecipitation (EZ-ChIP^™^) assay kit according to the manufacturer’s protocol (EMD Millipore). Anti-RNA polymerase II and normal mouse IgG were used as the positive and negative control antibody, respectively, for validating the ChIP assay. The epigenetic antibodies used in the ChIP assays were ChIP-validated acetyl-histone H3 (Upstate Biotechnology), acetyl-histone H4 (Upstate Biotechnology) and acetyl-histone H3 lysine9 (H3K9) (EMD Millipore). DNA was amplified by (rt) PCR using primers specific for the *ER*α promoter yielding a 150 bp fragment- forward: 5’-*GAACCGTCCGCAGCTCAAGATC*-3’ and reverse: 5’-*GTCTGACCGTAGACCTGCGCGTTG*-3’. Real-time PCR was performed using the SYBR green Supermix (Bio-Rad) and the reaction was initiated at 94°C for 10 min followed by 50 cycles of Denature: 94°C, 20 s; Anneal and extension: 60°C, 1 min. Fluorescence was detected by Roche Light cycler 480. Quantitative data was analyzed using percent input method for ChIP analysis (ThermoFisher Scientific), relative to vehicle control.

### HDAC activity assay

Cultured breast cancer cells were harvested at the indicated time points and nuclear extract was prepared using nuclear extraction reagent (Pierce, Rockford, IL). The HDAC activity assays were performed according to the manufacturer’s protocol (EpigentecK, Brooklyn, NY). The enzyme activity was detected by a microplate reader at 450 nM.

### HAT activity assay

Histone acetyltransferases (HAT) are a family of enzymes that transfer an acetyl group to histone protein. TNBC cells were harvested at the indicated time points and nuclear extract was prepared with the nuclear extraction reagent (Pierce, Rockford, IL). Overall HAT activity was determined using the EpiQuik HAT activity/inhibition assay kit (Epigentek) according to the manufacture’s protocol. This analysis provides the levels of overall HAT activity and is not specific to any particular gene or to any particular HAT, and data are represented in terms of percentage control.

### DNMT activity assay

DNA methyltransferases (DNMTs) are a group of enzymes that transfer a methyl group to the 5-carbon of the cytosine residue on DNA. Cells were plated in 6-well plates and treated with compounds for 72 h before extracting nuclear proteins (Pierce, Rockford, IL). Total DNMT activity was determined using EpiQuik DNMT Activity/Inhibition Assay Ultra Kit (Epigentek) according to manufacturer’s protocol.

### Methylated DNA (5-methylcytosine, 5-mC) quantification

For global analysis of DNA methylation, total genomic DNA was isolated from the cells after treating them at the indicated time points and different concentration of compounds alone and/or in combination. A total of 100 ng of DNA were used as the input. Overall DNA methylation was determined using MethylFlash Methylated DNA Quantification Kit (Colorimetric) according to the manufacture’s protocol. The quantification was detected by a microplate reader at 450 nM.

### Statistical analysis

All the data were determined from at least three independent experiments. Statistical significance of differences between the values of treated samples and control was determined by one way ANOVA using GraphPad Prism version 4.00 for Windows, graphPad Software (www.graphpad.com). In each case, p < 0.05 and p < 0.01 was considered statistically significant and highly significant, respectively.

## Results

### Combinatorial resveratrol and pterostilbene results in a time-dependent ERαreexpression in TNBC cell lines

**Fig 1:**
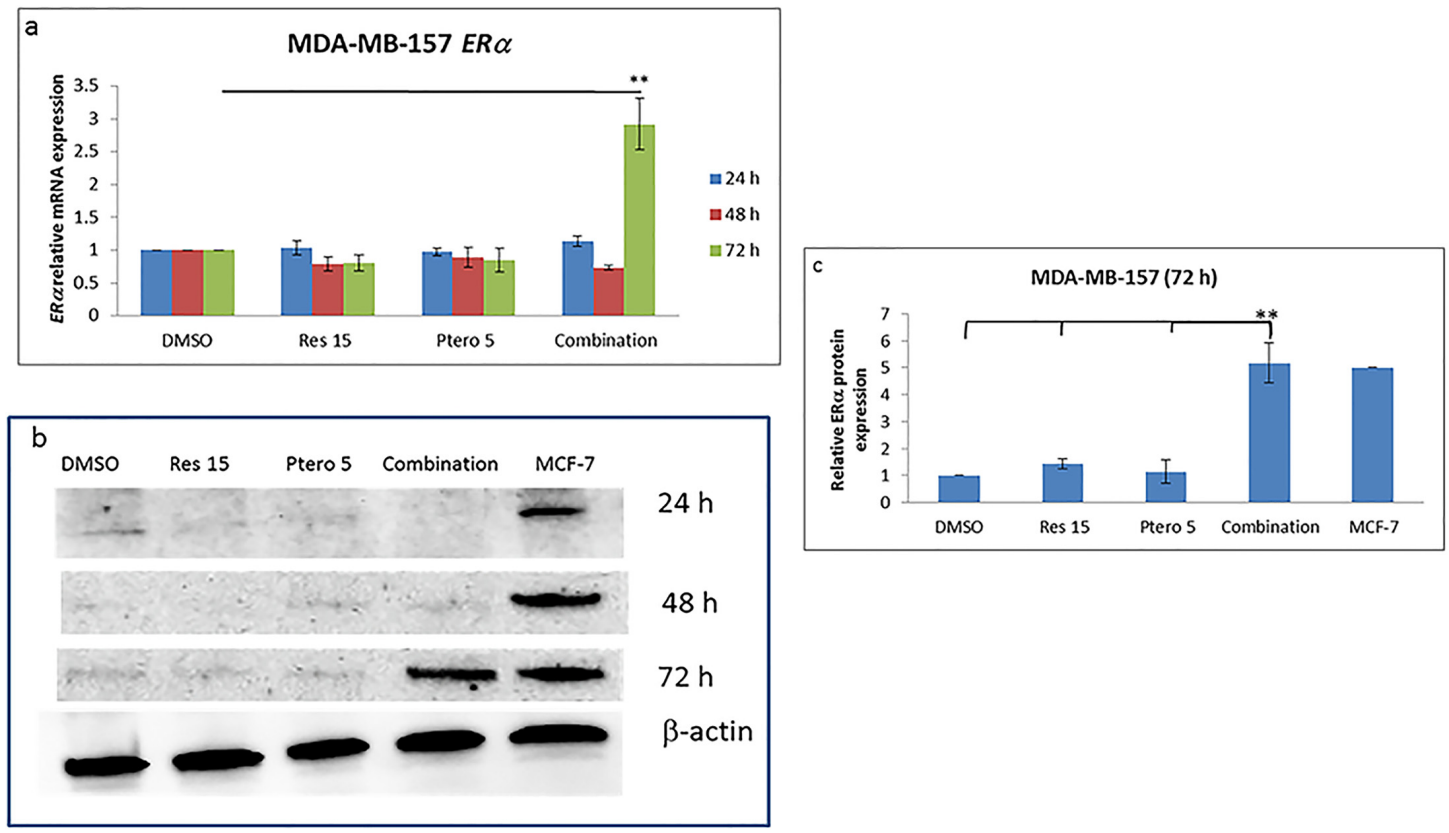
Combinatorial resveratrol and pterostilbene resulted in a time-dependent reexpression of ERαin MDA-MB-157 cells. **a)** Relative real-time *ERα*mRNA expression after 24, 48 and 72 h of treatments in MDA-MB-157 breast cancer cell lines. After 72 h of combinational treatment, there was a significant increase in *ERα* mRNA expression. GAPDH was used as the internal control. This increase in mRNA was found to be significant with all the treatments. **b and c)** Effects of compounds alone as well in combination on ERα protein expression after 24, 48 and 72 h of treatments. Fig b represents western blot at different time intervals and Fig c represents 72 h densitometry analysis of ERα protein expression at different treatments. Treatment for 72 h with compounds in combination in MDA-MB-157 cells resulted in a highly-significant increase in ERα protein levels. MCF-7 ERα protein extracts were used as the positive control and βactin was used as an internal control. Res 15, resveratrol 15 μM; Ptero 5, pterostilbene 5 μM; Combination, 15 μM resveratrol and 5 μM pterostilbene in combination. Values are representative of three independent experiments and are shown as percent of control ± SE; *P<0.05, **P<0.01.

**Fig 2:**
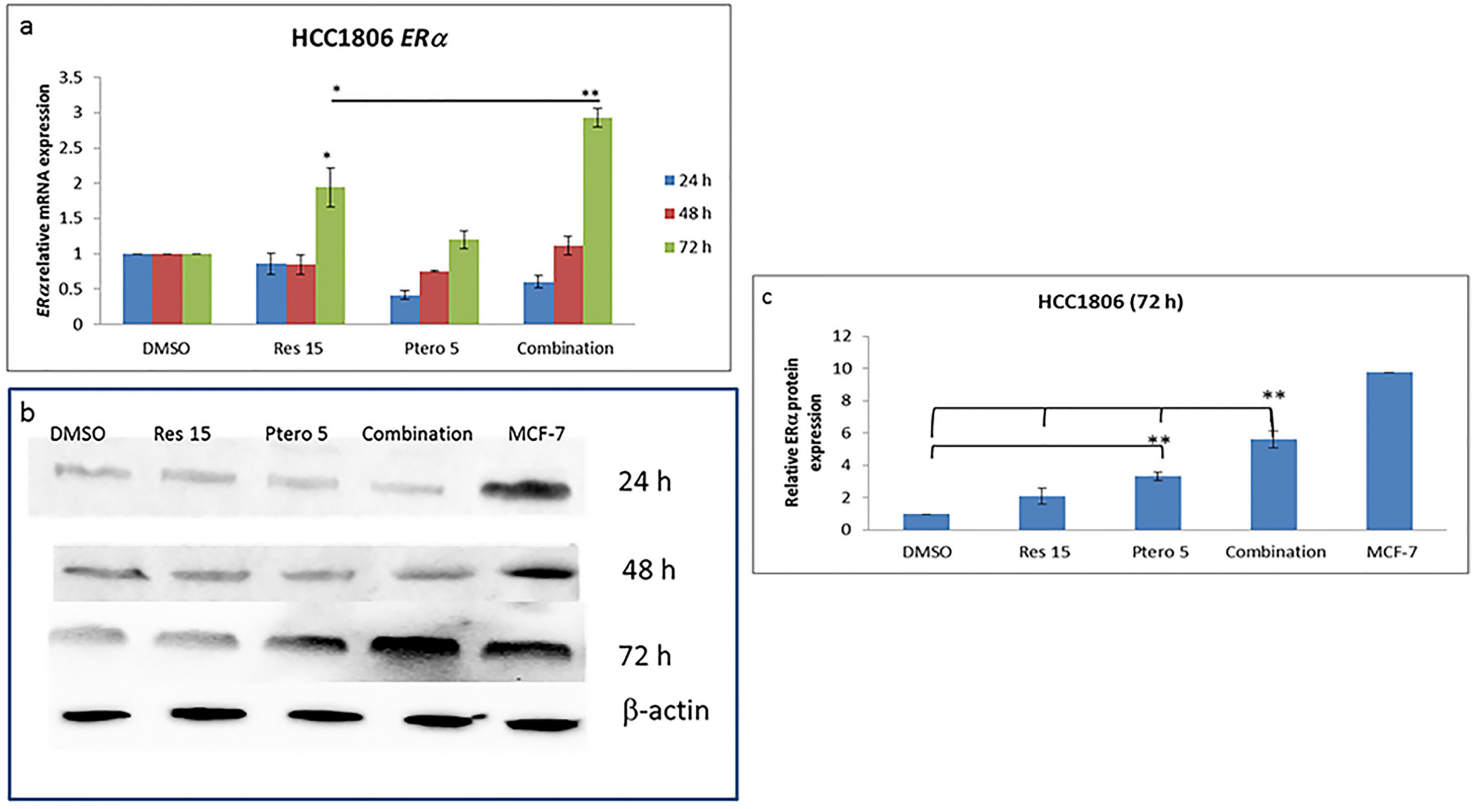
Combinatorial resveratrol and pterostilbene resulted in a time-dependent reexpression of ERα in HCC1806 cells. **a)** Relative real-time *ER*α mRNA expression after 24, 48 and 72 h of treatments in HCC1806 breast cancer cell lines. There was no significant effect of compounds alone or in combination at 24 h and 48 h of treatment; however, with combination treatment and 15 μM resveratrol after 72 h there was a significant increase in ERα mRNA expression. The increase with combination treatment was found to be more significant in comparison to other treatments at 72 h. GAPDH was used as the internal control. **b and c)** Effects of compounds alone as well as in combination on ERα protein expression after 24, 48 and 72 h of treatments. Fig b represents western blot analysis at three different time intervals and Fig c represents a densitometry analysis of ERα protein expression after 72 h. Treatment with pterostilbene as well as with combination of resveratrol and pterostilbene at 15 μM and 5 μM respectively, resulted in a significant increase in protein expression in HCC1806 cells after 72 h. This increase in expression with combination of compounds was found to be highly-significant when compared with other treatments. MCF-7 ERα protein extracts were used as the positive control and βactin was used as an internal control. Res 15, resveratrol 15 μM; Ptero 5, pterostilbene 5 μM; Combination, 15 μM resveratrol and 5 μM pterostilbene in combination. Values are representative of three independent experiments and are shown as percent of control ± SE; *P<0.05, **P<0.01.

To investigate time-dependent effects of resveratrol and pterostilbene on ERα reactivation, MDA-MB-157 and HCC1806 ERα-negative breast cell lines were treated for 24, 48 and 72 h, at an optimal dose of 15 μM of resveratrol and 5 μM of pterostilbene, singly as well as in-combination. As shown in Figs 1a and 2a for MDA-MB-157 (P<0.01) and HCC1806 (P<0.01) cells, respectively, 72 h of treatment results in a significant increase in *ERα* mRNA expression and this increase in expression was also confirmed at the protein level. Figs 1b and 2b display western blot analysis at different time intervals. Treatment with the compounds demonstrated no significant increase in ERαprotein expression at 24 h and 48 h as confirmed by western blots, but displays a significant increase in ERα protein expression at 72 h as shown in Figs 1b and 2b. Densitometry analysis (Figs 1c and 2c) at 72 h was performed to display the significant increase of ERα protein expression in both of the tested TNBC cell types. In MDA-MB-157 and HCC1806 breast cancer cells, combination treatment results in a highly significant (P<0.01) increase in ERα protein expression. In HCC1806 cells 5 μM pterostilbene single treatments also result in an increase in ERα expression, but combination treatments were found to be highly significant when compared with different treatment groups (Fig 2c). MCF-7 cell protein extract (Figs 1 and 2) were used as the positive control for ERα protein expression. This result indicates that the lower concentration of resveratrol and pterostilbene used in this study displayed a time-dependent reactivation of the ERα protein in the two TNBC cell lines. As published previously, these two compounds do not display any significant effects on cellular viability and apoptosis induction in MCF10A control cell lines and were found to possess synergy (CI<1) after 72 h of treatments in these two tested cell types [4]. Therefore, future experiments were performed with 15 μM resveratrol and 5 μM pterostilbene at indicated time points.

### Resveratrol combined with pterostilbene alters histone modification in the *ER*α promoter region in TNBC cells

**Fig 3:**
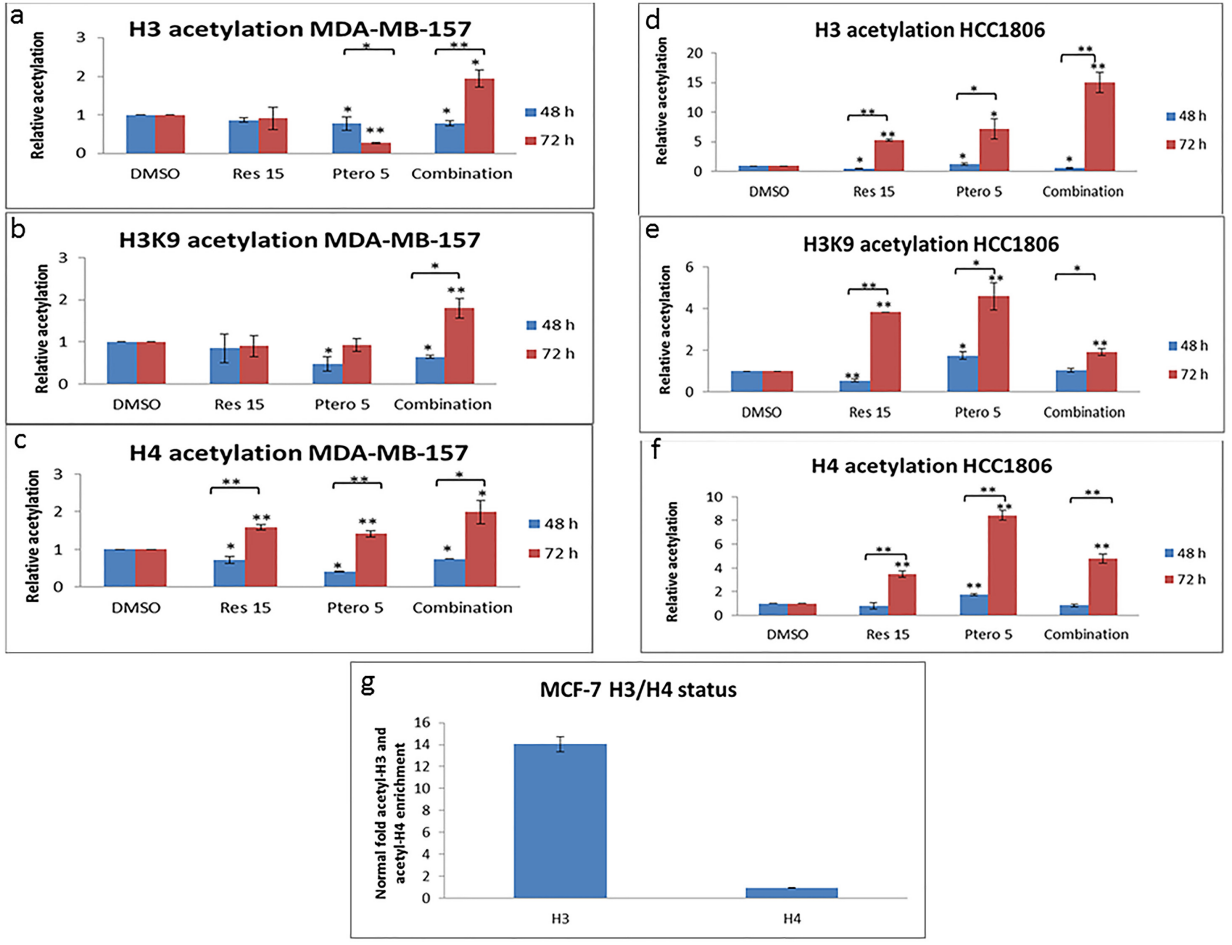
Combinational treatment results in an open chromatin structure in the *ER*α promoter region of TNBC cells. Histone modification enrichment in the *ERα* promoter was calculated from the corresponding DNA fragments amplified by ChIP-(RT) PCR. TNBC cells were treated as described previously and analyzed by ChIP assays using chromatin markers such as acetyl-H3, acetyl-H3K9 and acetyl-H4 for 48 h and 72 h. Percent input method for ChIP analysis-relative to control was used for analysis. **a and d)** Significant increase in acetyl-H3 enrichment in the *ERα* promoter region in both the tested cell lines after 48 to 72 h. MDA-MB-157 cells shown an increase enrichment of acetyl-H3 after 72 h of combination treatment which was highly significant when compared with DMSO, 48 h of treatment and 72 h of individual treatments. HCC1806 breast cancer cells (Fig 3d) shown an increase in acetyl-H3 enrichment with single doses at 72 h and pterostilbene 5 μM at 48 h, but the enrichment with combination of 15 μM resveratrol and 5 μM pterostilbene at 72 h was found to be more significant when compared within the treatment groups at both 48 h and 72 h in both the TNBC cell lines. **b and e)** Significant increase in acetyl-H3K9 enrichment at 72 h in the *ERα* promoter region in both of the tested cell types when compared with different groups at 48 h. However, in MDA-MB-157 cells the increase in H3K9 acetylation was found to be highly significant with combination of resveratrol and pterostilbene with no significant change within the 72 h treated group. Whereas, in HCC1806 breast cancer cells there was a significant increase with all the treatments groups at 72 h and 5 μM pterostilbene at 48 h when compared with DMSO control. **c and f)** A significant increase in acetyl-H4 enrichment in the *ERα* promoter region in both of the tested cell types with all the treatments when compared with DMSO group after 72 h. However, in MDA-MB-157 cells there was no significant difference found within the treatment group itself. In HCC1806 breast cancer cells there was a significant increase in acetyl-H4 enrichment within all the treatment groups after 72 h, but 5 μM pterostilbene at 48 h and 72 h when compared within the tested groups was found to be highly significant. **g)** Represents an open chromatin structure in the *ERα* promoter of ERα-positive MCF-7 breast cancer cells. Acetyl-H3 enrichment was found to be more predominant when compared with acetyl-H4 enrichment in MCF-7 cells. Res 15, resveratrol 15 μM; Ptero 5, pterostilbene 5 μM; Combination, 15 μM resveratrol and 5 μM pterostilbene in combination. Values are representative of three independent experiments and represented as percent of control ± SE; *P<0.05, **P<0.01.

Previous studies have shown that the ERα-negative breast cancer cells display a relative depletion of acetyl-H3 and acetyl-H4 histone markers in the promoter region, which provides a transcriptional repressive environment at the *ERα* gene [15]. Our current and previous studies have shown that combined treatment with resveratrol and pterostilbene at close to physiologically achievable doses significantly alters the activity and expression of histone modifying epigenetic machinery in both MDA-MB-157 and HCC1806 TNBC cells, suggesting a potential role of histone modification in *ERα* regulation. Both the ERα-negative breast cancer cell types were treated at the optimum dose of 15 μM resveratrol and 5 μM pterostilbene alone as well as in combination at these doses for 48 and 72 h. We found a significant increase in enrichment of acetyl-H3, acetyl-H3K9 and acetyl-H4 active chromatin marker between 48 h and 72 h in both of the cell lines (Fig 3). The acetyl-H3 enrichment with the combinatorial approach was significant in both MDA-MB-157 and HCC1806 breast cancer cells when compared to single-dose treatment, DMSO control and 48 h treatments. In case of H3K9 acetylation, there was an overall increase after 72 h treatments in MDA-MB-157 cells, which was highly significant when compared to single-dose of compounds, DMSO and 48 h of treatments (Fig 3b). In HCC1806 TNBC cell line there was an overall increase in acetyl-H3K9 enrichment between 48 h to 72 h of single doses and combination treatment (Fig 3e), even though the combination at 72 h was slightly low than the individual compounds, it was nevertheless significantly increased when compared with the DMSO control. In the case of acetyl-H4 enrichment, after 72 h of treatment there was a significant overall increase amongst all the treated groups in both of the breast cancer cell types; however, pterostilbene at 5 μM in HCC1806 cells was found to be highly significant when compared with individual treatments at 48 h and 72 h (Fig 3c and 3f). To further strengthen this study, MCF-7 ERα-positive cells were used as a positive marker for open/active chromatin acetyl-H3/H4 markers. Interestingly, acetyl-H3 chromatin marker (Fig 3g) were more prevalent than acetyl-H4 chromatin marker in MCF-7 cells, which further matches to our finding with acetyl-H3 and acetyl-H4 markers in both of the tested cell types in response to combinatorial approach after 72 h of treatments.

### Resveratrol and pterostilbene in combination alters epigenetic enzymes activity with no significant effects on MCF10A control cells

**Fig 4:**
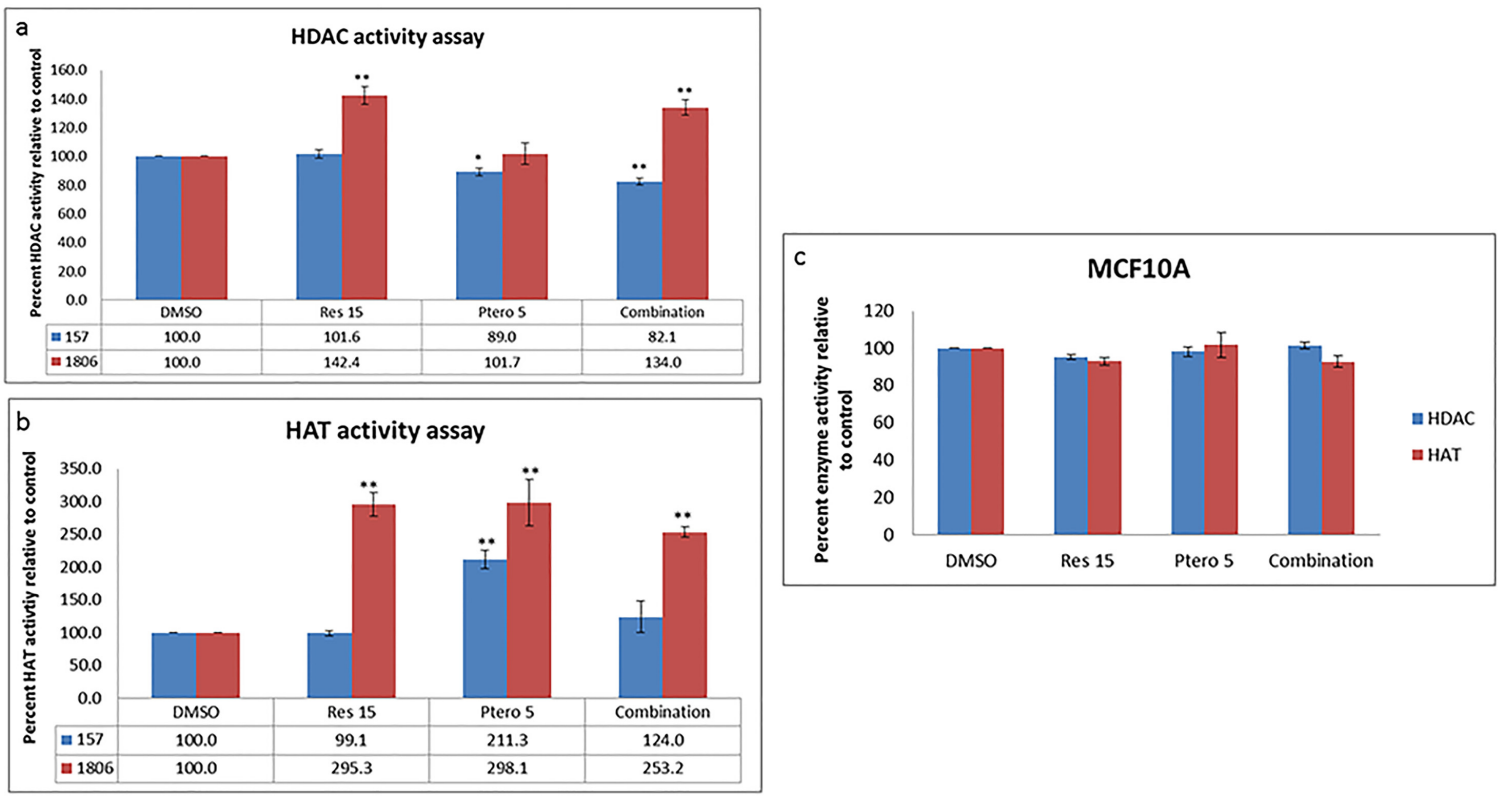
Resveratrol and pterostilbene alters epigenetic enzymes activity with no significant effects on MCF10A control cells. **a)** Overall HDAC percent enzyme activity relative to DMSO control was analyzed after 72 h of treatments in MDA-MB-157 and HCC1806 breast cancer cells using 20 μg of nuclear extract. After 72 h, 5 μM pterostilbene and combination treated group results in a significant down-regulation of enzyme activity in comparison to control in MDA-MB-157 cells. However, in HCC1806 cells there was a significant increase in overall enzyme activity with 15 μM resveratrol and combination treatments, but no significant change with 5 μM pterostilbene treatment. **b)** Overall HAT percent enzyme activity relative to DMSO control was analyzed after 72 h of treatments in MDA-MB-157 and HCC1806 breast cancer cells using 20 μg of nuclear extract. After 72 h, 5 μM pterostilbene results in a significant increase in overall enzyme activity with no significant change in any of the other treatment groups in MDA-MB-157 cells. Whereas, in HCC1806 cells there was a significant increase in overall enzyme activity in all the treatment groups, but no significant change within the treatment groups itself was found. **c)** Overall HDAC and HAT enzyme activities in MCF10A control cells. After 72 h, there was no significant change in the enzyme activity in comparison to DMSO control in MCF10A control cells further highlighting the effectiveness of this combination regimen. Res 15, resveratrol 15 μM; Ptero 5, pterostilbene 5 μM; Combination, 15 μM resveratrol and 5 μM pterostilbene in combination. Values are representative of three independent experiments and represented as percent of control ± SE; *P<0.05, **P<0.01.

To further explore the mechanism of ERα reexpression, we investigated the effects of compounds alone as well as in combination on HDAC and HAT activity. Fig 4a displays the overall percent HDAC activity relative to DMSO vehicle control after 72 h of treatments. Interestingly, MDA-MB-157 cells show a significant decrease in HDAC activity with 5 μM pterostilbene (P<0.05) and with combination treatments (P<0.01) when compared to control after 72 h. On the other hand, HCC1806 cells display a significant (P<0.01) increase after 72 h of treatment with 15 μM resveratrol and compounds in combination. Both the compounds were also tested for their effects on HAT activity as shown in Fig 4b. Ironically, MDA-MB-157 cells exhibit an increase in HAT activity after 72 h of treatment with 5 μM of pterostilbene but no significant change in activity after single dose of resveratrol 15 μM and combination treatments. However, there is a significant increase in HAT activity with all the treatments in HCC1806 TNBC cells (Fig 4b), with no significant difference within the treated groups. These changes were roughly around 2-fold increase when compared with the control. The compounds alone as well as in combination after 72 h of treatment did not display any significant changes in percent HDAC and HAT enzyme activity in MCF10A control cells (Fig 4c), thereby indicating the efficacy of this combinatorial dietary approach in TNBC cancer cells, with no significant effects on control breast epithelial cells.

### Combinatorial treatment results in global change in 5-methylcytosine (5-mC) and DNMT enzyme activity (global DNA methylation patterns)

**Fig 5:**
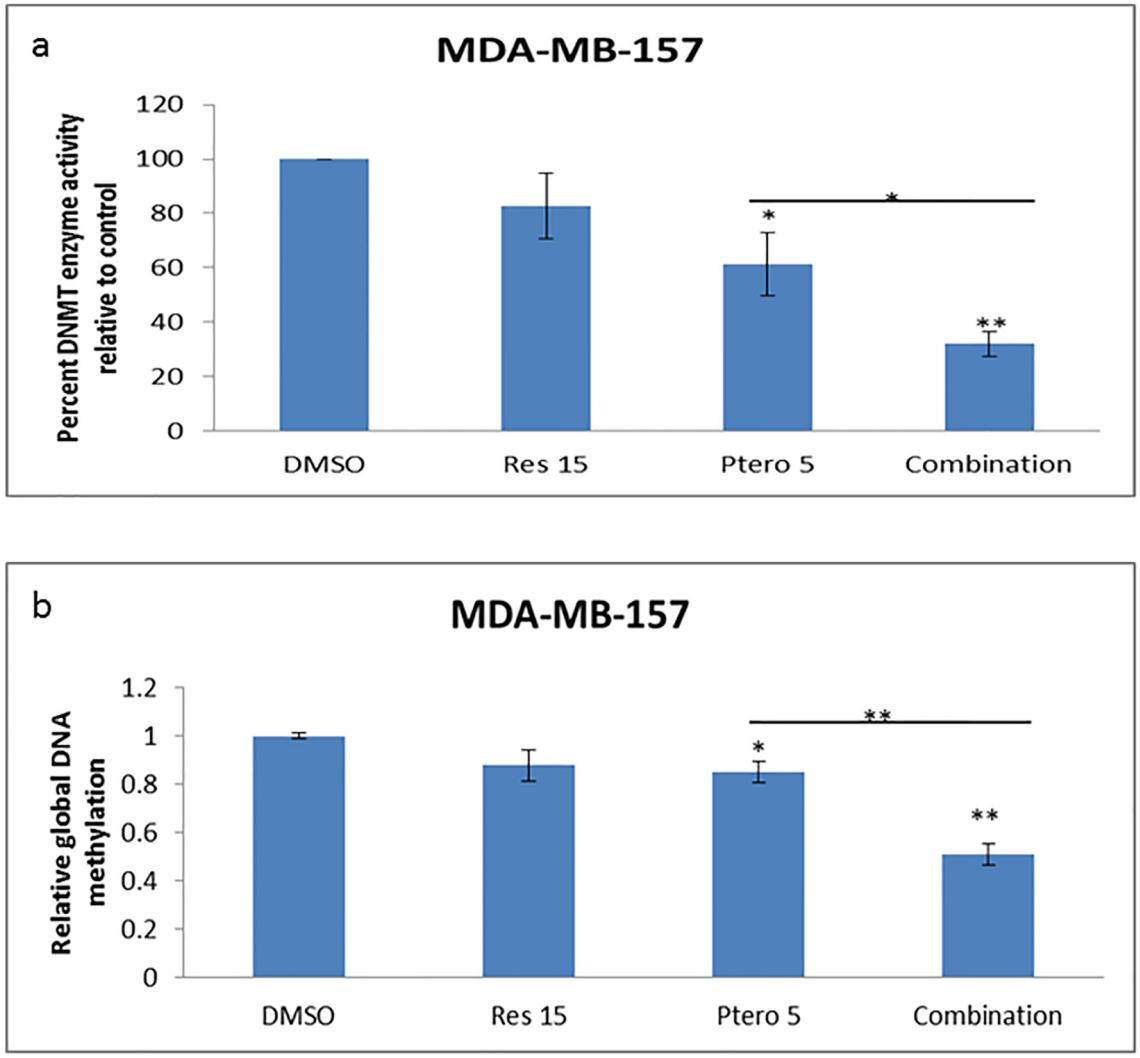
Combinatorial treatment results in a significant decrease in DNMT enzyme activity and global DNA methylation. Fig 5a shows a relative decrease in overall DNMT enzyme activity with 5 μM pterostilbene and combination treatments. These decreases in DNMT activity with combination were found to be highly significant when compared with different treatment groups. Fig 5b shows a relative decrease in global DNA methylation pattern with 5 μM pterostilbene and combination treatments. This decrease in 5-mC levels with combination was found to be highly significant and in accordance to our DNMT data, when compared with different treatment groups. Res 15, resveratrol 15 μM; Ptero 5, pterostilbene 5 μM; Combination, 15 μM resveratrol and 5 μM pterostilbene in combination. Values are representative of three independent experiments and represented as relative of DMSO control ± SE; *P<0.05, **P<0.01.

Recent studies have shown that predominantly histone modification and to some extent DNA methylation can contribute to *ERα* regulation [6, 7, 16]. Our previous work with these two compounds in HCC1806 breast cancer cells has documented a significant decrease in overall DNMT enzyme activity, with no significant effects on control MCF10A DNMT enzyme activity after 72 h of treatments [4]. Therefore, we felt it was important to test the compounds for their effects on global changes in 5-methylcytosine levels as well as DNMT enzyme activity in MDA-MB-157 breast cancer cells. Since few differences were observed between the two tested cell lines, we next focused on MDA-MB-157 cells to explore more in depth the mechanism for this observed ERα reactivation. MDA-MB-157 cells were treated for 72 h at an optimal dose of 15 μM resveratrol and 5 μM pterostilbene alone as well as in combination. Overall DNMT enzyme activities were performed using a total of 20 μg of nuclear protein as the starting material. Fig 5a shows a significant decrease in overall DNMT enzyme activity after 5 μM pterostilbene and combination treatments. The combination treatment displays a significant (P<0.05) decline in DNMT enzyme activity when compared with individual treatments and DMSO vehicle control cells after 72 h. To further investigate the effects of this decrease in DNMT enzyme activity on global DNA methylation patterns, total DNA was extracted and 100 ng of input DNA was used as the starting material. Fig 5b shows a significant decrease in global DNA methylation (5-mC levels) after 5 μM pterostilbene and combination treatments. The combination treatment displayed a highly significant (P<0.01) decline in DNA methylation when compared with individual treatments and DMSO vehicle control after 72 h. This change in 5-mC pattern were found to be in accordance to our DNMT data and could also account for this *ERα* reactivation in TNBC cells after combinatorial treatments.

### Combinatorial resveratrol and pterostilbene sensitize ER-negative breast cancer cells to E2 and 4-hydroxytamoxifen (4-OHT) treatment

**Fig 6:**
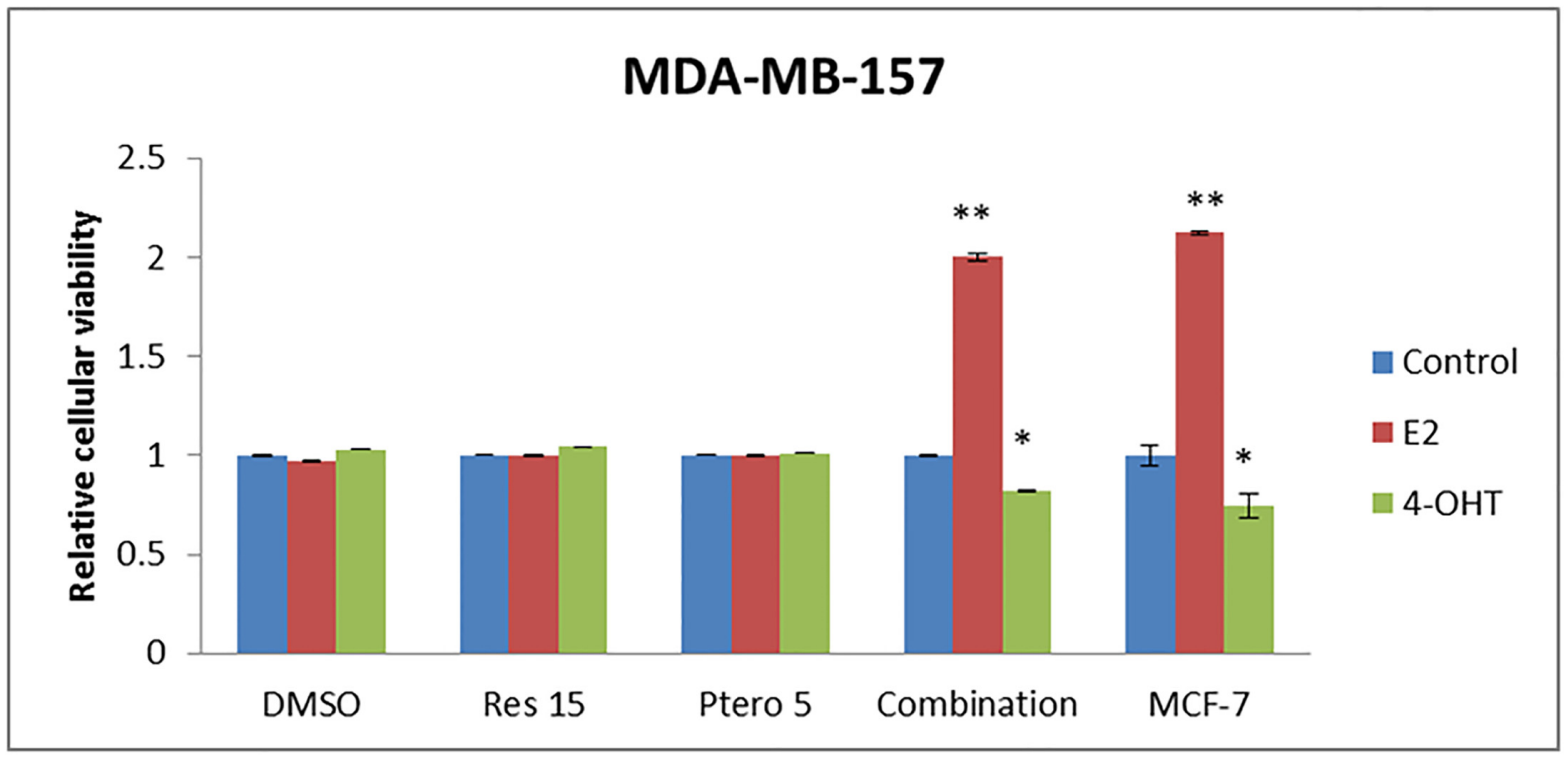
Combinatorial treatments result in sensitizing ERα-negative cells towards E2 and 4-hydroxytamoxifen (4-OHT) treatments. MDA-MB-157 cells were treated with or without 10 nM E2 or 5 μM 4-OHT for 48 h after 72 h of treatment with the dietary compounds. MCF-7 cells served as a positive control. Cells without any E2 or 4-OHT served as the system control within the different treatment sets. Interestingly, with combination treatment and E2 (ligand activator) there was a significant increase in cellular viability and, with 4-OHT (antagonist) there was a significant decrease in cellular viability, as analyzed by MTT assay. MCF-7 cells when treated with E2 (ligand activator) and 4-OHT (antagonist) for 48 h showed a similar responsiveness. Res 15, resveratrol 15 μM; Ptero 5, pterostilbene 5 μM; Combination, 15 μM resveratrol and 5 μM pterostilbene in combination. Values are representative of three independent experiments and represented as relative to control ± SE; *P<0.05, **P<0.01.

So far, our aforementioned observations suggest that the combination of dietary resveratrol and pterostilbene epigenetically reactivate ERα expression in ERα-negative breast cancer cells at both the mRNA as well as protein levels in a time-dependent manner. To further strengthen our findings and explore clinical potential of this dietary approach, we investigated ERα-dependent cellular responsiveness either to the ligand activator, 17β-estradiol (E2), or to the antagonist, 4-hydroxytamoxifen (4-OHT; active metabolite of tamoxifen) in MDA-MB-157 cells. MCF-7 ERα-positive cells were used as the positive control cells. MDA-MB-157 cells were first treated with 15 μM resveratrol or 5 μM pterostilbene or in combination for 72 h followed by treatment with E2 and 4-OHT for 48 h in order to determine effects on cellular viability. Fig 6 shows a highly significant (P<0.01) increase in cellular proliferation after combination (72 h) and E2 (next 48 h) treatments and a significant (P<0.05) decrease in cellular proliferation after combination (72 h) and 4-OHT (next 48 h) treatments. When treated with compounds singly with and/or without E2 and 4-OHT, no significant changes in cellular proliferation were observed (Fig 6). This could be due to limited increase in the ERα expression which were similar to our previous experiments. MCF-7 cells were treated with E2 and 4-OHT for 48 h and displayed a similar trend of significance (Fig 6).

### E2 and 4-OHT further affected ERα-responsive downstream *progesterone receptor* (*PGR*) gene in ERα resensitized MDA-MB-157 TNBC cells

**Fig 7:**
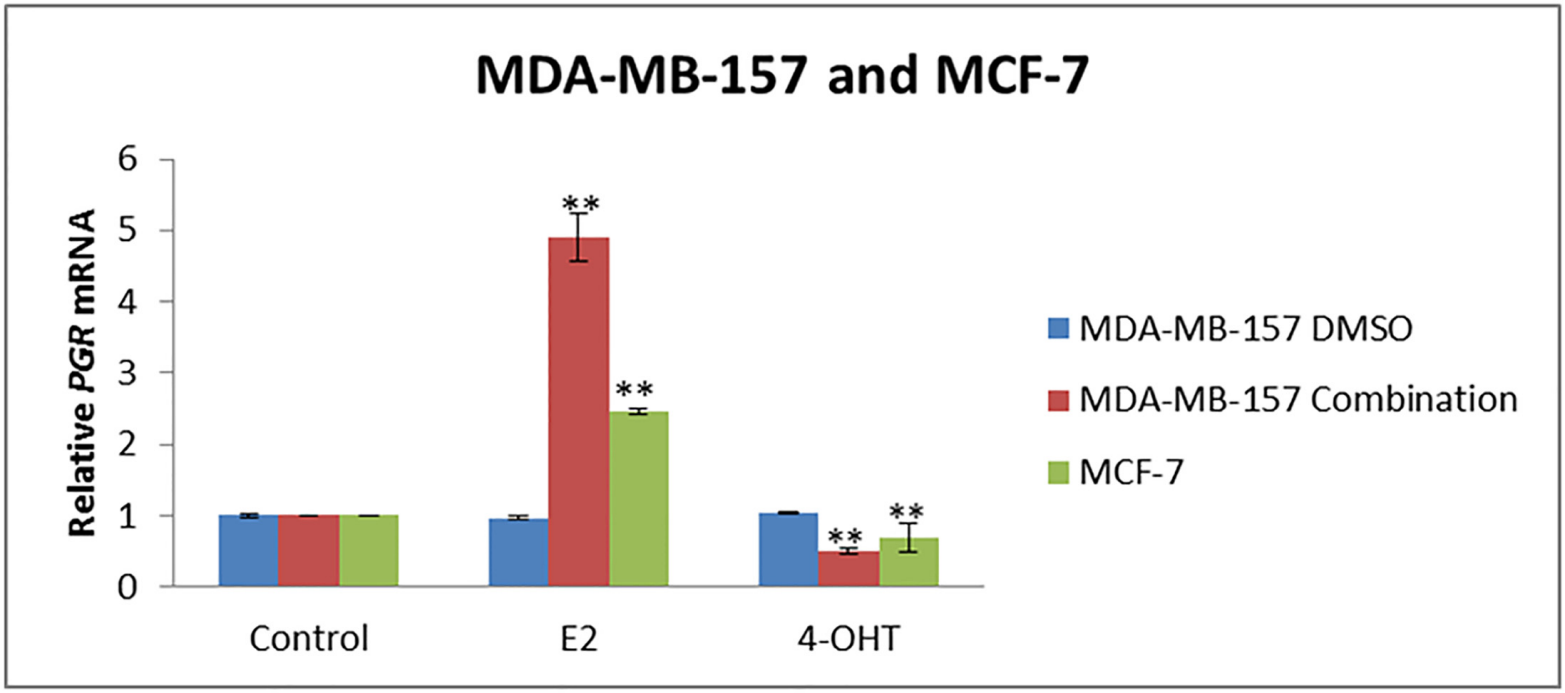
Combinatorial treatment retrieved responsiveness to E2 and 4-hydroxytamoxifen (4-OHT) treatments in resensitized MDA-MB-157 TNBC cells. *PGR* expression was detected by quantitative real-time PCR. GAPDH was used as the internal control. MDA-MB-157 cells were treated with or without 10 nM E2 or 5 μM 4-OHT for 48 h after 72 h of treatment with combinatorial treatment. MCF-7 cells served as a positive control. Cells without any E2 or 4-OHT served as the system control within the different treatment sets. Combination treatment along with E2 (ligand activator) treatment resulted in a significant increase in *PRG* mRNA and, with 4-OHT (antagonist) treatment, resulted in a significant decrease in *PGR* mRNA expression. MCF-7 cells when treated with E2 (ligand activator) and 4-OHT (antagonist) for 48 h showed a similar *PGR* mRNA responsiveness. Values are representative of three independent experiments and are relative to control ± SE; *P<0.05, **P<0.01.

To further confirm the ERα reactivation after combination treatments in MDA-MB-157 cells, we investigated the effects on ERα downstream gene target such as *PGR*, with treatments of E2 and 4-OHT following combinational treatments (72 h). ERα positive MCF-7 cells served as the positive control. As shown in Fig 7, MDA-MB-157 cells display a significant (P<0.01) increase in *PGR* mRNA response with E2 treatment after combinatorial treatments. However, 4-OHT treatment display a significant (P<0.01) decrease in *PGR* mRNA followed after combinatorial approach. MCF-7 cells display a similar *PGR* response trend after 48 h of treatments with E2 and 4-OHT.

## Discussion

Current clinical prognosis and treatment strategies for malignant breast cancers rely in part on ERαexpression, being that patients with an ERα-positive status respond better to available endocrine therapy, such as tamoxifen. However, the patients with an ERα-negative status lack this opportunity and are often resistant to anti-hormonal therapies and the cancers are more aggressive and confer poorer prognosis [17, 18]. Since ERα-negative tumors are difficult to treat with available endocrine therapy, it is crucial to formulate new strategies to sensitize ERα-negative tumors to available endocrine treatments.

Epigenetic-based therapies have attracted considerable excitement over the past few years. To date, many studies have shown that the use of 5-aza, a DNMT inhibitor, and TSA, a HDAC inhibitor, can reactivate the expression of *ERα* in ERα-negative breast cancer cells, further strengthening the role of epigenetic mechanisms in regulating and driving cancers [11, 19]. However, the use of synthetic molecules or analogs of compounds might result in adverse side effects. Therefore, the use of bioactive dietary compounds with a potential to inhibit DNMT and HDAC activity and/or increase HAT activity could ideally be used for ERα reactivation. This could be of great benefit in terms of overall health, reduced treatment cost, and a more natural dietary regimen. Resveratrol and pterostilbene are bioactive dietary phytochemicals which have been documented to affect DNA methylation and histone acetylation by targeting DNMTs and SIRT1 (HDACIII) activity, respectively [4, 20,21].

In the current study, we analyzed the effects of combinatorial 15 μM resveratrol and 5 μM pterostilbene in reactivating endogenous ERα expression in both of the tested TNBC cell lines. This reactivation is reported for the first time with these two compounds in combination on MDA-MB-157 and HCC1806 breast cancer cells. This conversion of ERα-negative to ERα-positive status could be due, in part, to epigenetic changes in global DNA methylation and chromatin modification of the *ERα* gene promoter. Resveratrol 15 μM and pterostilbene 5 μM when administered (Figs 1 and 2), displayed a time-dependent reactivation of ERα status in the tested cell types. The concentrations used in this study have been previously tested in the control MCF10A breast non-tumorigenic epithelial cell lines for toxicity and efficacy. So far the concentration of both of the dietary compounds is found to inhibit cell growth (CI<1) synergistically [4] and reactivate ERα expression at 72 h in these two tested TNBC cell lines.

To further understand the mechanism behind this reactivation of ERα in TNBC cells, we performed time-dependent ChIP analysis to investigate combined resveratrol and pterostilbene-induced enrichment of transcriptional active chromatin markers such as acetylated-histone H3, acetyl-H3K9 and acetyl-H4 in ERα-negative MDA-MB-157 and HCC1806 breast cancer cells. Previous studies with dietary compounds such as sulforaphane (SFN) and epigallocatechin gallate (EGCG) have documented an increased enrichment of histone active markers within the *ERα* promoter of ERα-negative cells [6, 7], which resulted in an open/more active chromatin structure. The present study is the first study to address these effects with these two dietary compounds.

Interestingly, in the cell lines tested there was an increased enrichment of acetyl-H3, acetyl-H3K9 and acetyl-H4 markers in *ERα* promoter between 48 h to 72 h of resveratrol and pterostilbene treatments (Fig 3). Although there was a significant increase in acetyl-H3 and acetyl-H3K9 enrichment with combination treatments after 72 h in MDA-MB-157 cells (Fig 3a and 3b), there was no significant difference in acetyl-H4 enrichment within the treatment group after 72 h in MDA-MB-157 cells (Fig 3c). Nevertheless, these changes were highly significant when compared with the DMSO and 48 h treatment groups. Whereas in HCC1806 there was a significant increase in acetyl-H3, acetyl-H3K9 and acetyl-H4 after 72 h of treatments, the combination in acetyl-H3 was found to be highly significant when compared within the treatment groups (Fig 3d). Although there was a slight decrease in acetyl-H3K9 and acetyl-H4 enrichment after 72 h of combination treatment in comparison to 5 μM of pterostilbene (Fig 3e and 3f), overall the treatments were found to be highly significant when compared with the DMSO group. This increased enrichment of acetyl-H4 and acetyl-H3K9 could account for a slight increase in ERα protein expression at 5 μM of pterostilbene in Fig 2b after 72 h. Also, the acetyl-H3 enrichment in HCC1806 cells after 72 h of combination treatment was ~1.88 fold more than H4 and ~3 fold more than H3K9 acetylation after 5 μM pterostilbene treatment, which could account for the overall increase in ERα protein expression in Fig 2b. This change in acetylation status of histones should have resulted in an open chromatin structure in the *ERα*promoter which might result in release of co-repressor complex, as established previously in our lab [6, 7, 15]. ChIP analysis of MCF-7 breast cancer cells displayed an abundance of active acetyl-histone H3 and acetyl-H4 chromatin markers in the *ERα* core promoter region, which further matches to previous published *ERα* promoter acetyl-H3 and/or H4 enrichment data in ER*α*-positive breast cancer cells [6, 7, 16]

To further understand the mechanisms of epigenetic modulation of combined resveratrol and pterostilbene treatment on ERα reactivation in these two cell types, we assessed the histone acetylation-related enzymatic activities at 72h. Since there were no significant changes observed in histone acetylation or ERα protein expression after 48 h of treatments in both of the cells lines, all subsequent experiments were performed at 72 h. Previous work with these two compounds in combination on MDA-MB-157 and HCC1806 breast cancer cells have shown them to affect the activity of SIRT1; therefore, we investigated the effects of compounds on HDAC and HAT enzyme activities in the current study. Together our results in MDA-MB-157 cells have shown a significant decrease in HDAC enzyme activity with combination and pterostilbene 5 μM treatments (Fig 4a). Fig 4b displays a significant increase in HAT enzyme activity with 5 μM pterostilbene and no significant increase with combinational and resveratrol 15 μM treatments. However, it was interesting to see an increase in HDAC enzyme activity in HCC1806 with combination and resveratrol 15 μM treatments (Fig 4a), along with a significant increase in HAT enzyme activity within all the treatment groups (Fig 4b). This decrease in HDAC enzyme activity in MDA-MB-157 cells and an increase in HAT activity in HCC1806 cells, along with documented effects on SIRT1 in both the cancer cell lines [4] together may account for a change in histone acetylation at the *ERα* promoter region. Recent studies targeting SIRT1 protein expression have reported an increase in acetylation of histone-H3 and H4 in the promoter region, resulting in more open chromatin structures [22–24]. To further prove the effectiveness and efficacy of this dietary regimen, HDAC and HAT activity assays were performed in MCF10A control breast epithelial cells. No significant differences were observed within the treatment groups (Fig 4c), which supports the safety and efficacy of this dietary regimen and is in accordance with our previously published data with MCF10A cells [4].

Studies have shown that increase in CpG methylation and DNMT activity in the genome results in silencing of several tumor suppressor genes such as *p16*, *ER* and *PTEN* in cancer cells [7, 25–27]. Our work with 15 μM resveratrol and 5 μM pterostilbene in MDA-MB-157 cells (Fig 5) demonstrates a significant decrease in DNMT enzyme activity which in turn might have affected overall DNA methylation patterns by decreasing 5-mC levels in the CpG sites globally. In the genome, approximately 80% of the 5-mC residues are found within CpG dinucleotides, which accumulate to form CpG islands [28]; hence this change in 5-mC levels might directly impact global CpG islands methylation pattern. Taken together, it is apparent that a decrease in DNMT activity, 5-mC levels and HDAC/HAT-associated chromatin remodeling might have resulted in a more open chromatin structure, which may be linked to *ERα*reactivation by resveratrol and pterostilbene in these two ERα-negative human breast cancer cell lines. So far, our current results and past results [4] with these two compounds in MDA-MB-157 and HCC1806 breast cancer cells have shown impact on the epigenetic modifying machinery at a concentration which is, so far, found to be safe and close to physiologically achievable doses. This could also play a significant role in reshaping the cancer epigenome as these epigenetic modifying enzymes are often dysregulated in cancer and are current targets for cancer therapies.

After determining the possible mechanism of ERα reactivation in the tested cell types, we further investigated the functional ERα reactivation by testing them against conventional tamoxifen hormonal therapy. MDA-MB-157 cells were first treated with the combined compounds for 72 h and then treated with E2 (ligand activator) or 4-Hydroxytamoxifen 4-OHT (antagonist) for 48 h at a concentration of 10 nM and 5 μM, respectively. The role of E2 is to promote cellular proliferation through ligand-receptor activation and the role of 4-OHT (an active metabolite of tamoxifen) is as an antagonist by competing with estrogen for binding to functional ER, which will eventually lead to cell growth arrest [6, 29]. Interestingly, and in accordance to our previous data, there was a significant increase in cellular proliferation in MDA-MB-157 cells after combination of 15 μM resveratrol and 5 μM pterostilbene (72 h) with E2 treatments (48 h). There was a significant decrease in cellular proliferation with 4-OHT (48 h) treatment followed after combinational (72 h) treatments. This change in cellular proliferation was compared with the combination of 15 μM resveratrol and 5 μM pterostilbene (72 h) alone, E2 and/or 4-OHT treatment groups. In addition to cellular viability, ERα-dependent downstream *PGR* gene expression was also analyzed in MDA-MB-157 cells after 72 h of combinational treatment followed by 48 h of E2 or 4-OHT treatments. As expected, this combination of dietary compounds with available anti-hormonal therapy resulted in a significant increase in *PGR* mRNA after E2 treatments and a significant decrease in *PGR* mRNA after 4-OHT treatments when compared with different treatment groups. ERα-positive MCF-7 cells display a similar trend after 48 h of E2 or 4-OHT treatments, respectively.

Hence, these findings indicate that the compounds, resveratrol and pterostilbene, can not only reactivate the expression of ERα in part through epigenetic modulation in TNBC cells, but can also sensitize them to respond to conventional anti-hormonal therapy, which can last for a while (48 h in this study) after the dietary compound-treatments are completed. In the future, this dietary regimen may provide a new therapeutic approach to treat TNBCs and/or allow administered as an adjuvant therapy with currently available tamoxifen treatment.

## Conclusion

Our results with combinatorial resveratrol and pterostilbene at a dose which is close to physiological-relevant concentrations not only significantly reactivates the ERα status via its effects on DNA methylation and histone acetylation in these ERα-negative breast cancer cells, but can also synergistically inhibit the viability of TNBC cells and decrease the expression of *hTERT* and SIRT1 (HDAC III), as published in our previous findings. The compounds have also been documented to enhance the cytotoxic effects of DNA damaging agent and irradiation therapy (IR) by targeting SIRT1-regulated γ -H2AX expression in TNBC cells, with no overall effects on control MCF10A breast epithelial cells. Together all these findings provide a promising future for these two dietary compounds in combination with a potential to target multiple pathways in affecting the growth of TNBC.

## Abbreviations

ERα: estrogen receptor-alpha
TNBC: triple-negative breast cancers
HDAC: histone deacetylases
HAT: histone acetyltransferases
DNMT: DNA methyltransferases
DDR: DNA damage response
5-mC: 5-methylcytosine
4-OHT: 4-hydroxytamoxifen
E2: 17β-estradiol
TSA: Trichostatin A
5-aza: 5-azacytidine
CI: combination index
